# A shell dataset, for shell features extraction and recognition

**DOI:** 10.1038/s41597-019-0230-3

**Published:** 2019-10-22

**Authors:** Qi Zhang, Jianhang Zhou, Jing He, Xiaodong Cun, Shaoning Zeng, Bob Zhang

**Affiliations:** 1 Department of Computer and Information Science, University of Macau, Taipa, Macau China; 2 Citizen Scientist, Sanxin Road (N) 1755/117, Songjiang, Shanghai China; 3 School of Information Science and Technology, Huizhou University, Avenue Yanda, No. 46, Huizhou, Guangdong China

**Keywords:** Zoology, Computer science

## Abstract

Shells are very common objects in the world, often used for decorations, collections, academic research, etc. With tens of thousands of species, shells are not easy to identify manually. Until now, no one has proposed the recognition of shells using machine learning techniques. We initially present a shell dataset, containing 7894 shell species with 29622 samples, where totally 59244 shell images for shell features extraction and recognition are used. Three features of shells, namely colour, shape and texture were generated from 134 shell species with 10 samples, which were then validated by two different classifiers: *k*-nearest neighbours (*k*-NN) and random forest. Since the development of conchology is mature, we believe this dataset can represent a valuable resource for automatic shell recognition. The extracted features of shells are also useful in developing and optimizing new machine learning techniques. Furthermore, we hope more researchers can present new methods to extract shell features and develop new classifiers based on this dataset, in order to improve the recognition performance of shell species.

## Abstract (summary)

**Table:** 

Measurement(s)	shell • Taxonomy
Technology Type(s)	digital curation • machine learning
Factor Type(s)	shape • colour • texture • species

Machine-accessible metadata file describing the reported data: 10.6084/m9.figshare.9939353

## Background & Summary

In human history the utilization of shells has occurred for thousands of years. The cowrie shells are commonly found in Bronze Age sites in ancient China, and usually regarded as money or currency during the Shang and Zhou periods^1^. In Western Europe, the Sowerby family was active and presented numerous works on molluscs, and its systematics from the late eighteenth century to mid twentieth century. According to statistics, the Sowerby family introduced the names of more than 2000 shell species and produced many books on the genera of shells^2,3^.

Today shell collection and the development of conchology are on the uprise. The Sanibel Shell Festival has been held consecutively more than 70 years^4^. Meanwhile, many academic books or journals about shell research and classification have recently gained more popularity. A publisher called ConchBooks that specializes in shell research, has published more than 3000 books about shell research (https://www.conchbooks.de/?t=1).

Although there are so many works on shell collection and identification, it is still difficult to recognise shell species manually, as shells have tens of thousands of classes^5,6^. Thus, this problem indeed hampers the passion of shell collecting amateurs and the development of conchology. With the growth of the Internet and the progress of artificial intelligence^7^, it is possible and useful to investigate shell classification using machine learning techniques.

**Fig. 1:**
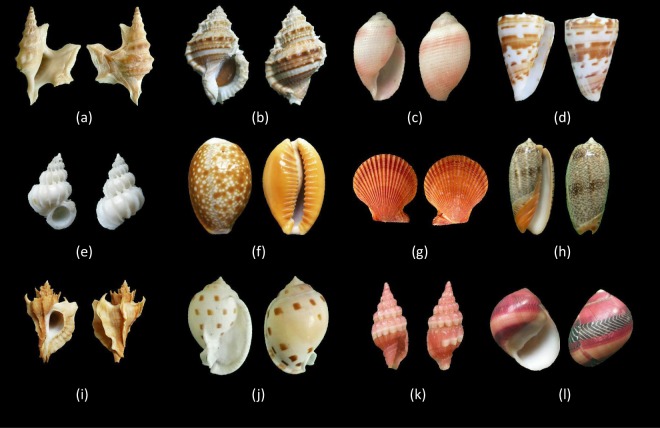
A small variety of shell species that are part of this shell dataset. From top to bottom are (**a**) Aporrhais pespelicani, (**b**) Bufonaria nana, (**c**) Bullina virgo, (**d**) Conus advertex, (**e**) Epitonium tokyoense, (**f**) Erosaria helvola, (**g**) Mimachlamys asperrima, (**h**) Oliva reticulata, (**i**) Pteropurpura adunca, (**j**) Semicassis bisulcate, (**k**) Vexillum rubrum, (**l**) Vittina waigiensis. Each shell sample contains two images of the frontal and lateral, and all shell samples are organized carefully with the photos taken based on this rule.

In this article we present a large shell dataset, containing 7894 shell species with 59244 shell images. Each species has shell samples ranging from 1 to 87 respectively, and every shell sample has two photographs taken at different views: frontal and lateral by us (Fig. 1). As different shells have different colours, shapes and decorative patterns, which can be used to identify shell species by artificial intelligence, three shell features: colour, shape and texture were generated from shell sample images by some image processing methods. Two classifiers: *k*-NN^8^ and random forest^9^ were applied for evaluating the extracted shell features. Preliminary experiments in the technical validation section with positive results show the potential and effective capability of using this data successfully in automatic shell recognition.

We hope more researchers especially computer scientists attempt to re-use this shell dataset, propose novel feature extraction methods or new classification methods to improve the performance of shell recognition. Since this work just extracts three common features of a shell, some special features such as geometric patterns are not investigated^10^. The extracted features from shells are also useful for developing and optimizing new machine learning techniques. Due to the fact that only two simple classifiers: *k*-NN and random forest were used in this article to evaluate the shell classification performance, many state-of-the-art machine learning methods such as convolutional neural network^11,12^ may be reasonable ways to access shell classification results in the future.

## Methods

**Fig. 2:**
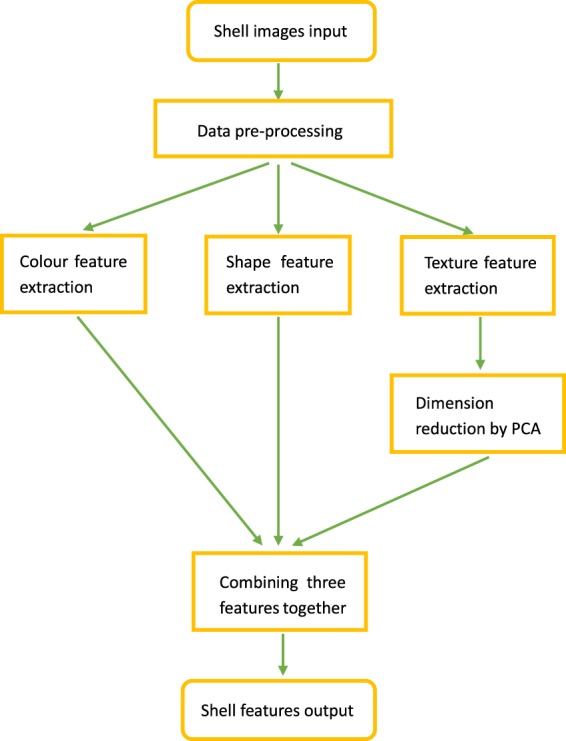
The flow diagram of generating shell features.

In this work we first collected and reorganized the shell images. Afterwards, three shell features were extracted from the shell dataset by applying some image processing methods. These extracted features will be validated by two classifiers in the technical validation section to prove the quality of this shell dataset. Fig. 2 shows the procedures of generating shell features in this dataset.

### Data Pre-processing

Our shell data collection contains 7894 shell species with 29622 samples, where each sample has two different views of colour images (JPG format). Each shell image was labelled with its scientific name and corresponding number, then resized to 300*400 pixels to be further processed to generate its features.

### Colour feature extraction

**Fig. 3:**
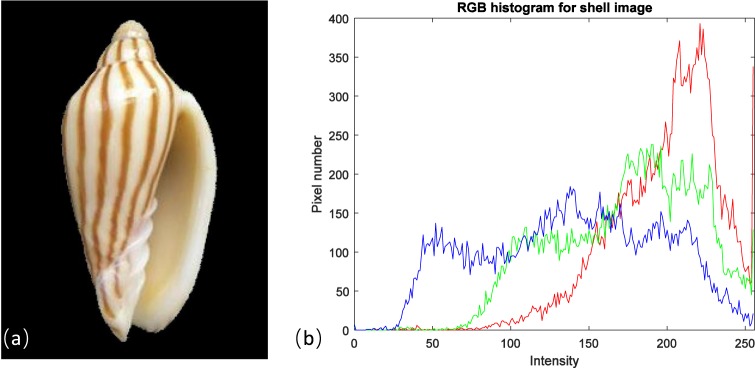
The RGB histogram generated from a shell image. Figure (**a**) indicates a colour shell image (scientific name: Amoria dampieria, front view, image size: 300*400, ID number in shell dataset: Amoria_dampieria_10_A), while plot (**b**) shows its corresponding colour histograms (red, green and blue curves) for the red, green and blue channels of this shell.

Since colour feature extraction from shells has not been investigated before, we can refer to some leaf and flower recognition works, as leaf, flower and shell have different colours on their surface. Caglayan *et al*. applied three histograms to the red, green and blue channels of leaf images, before calculating the mean and standard deviation in each histogram as the colour features for classifying the Flavia dataset^13^. Mishra *et al*. used a RGB histogram to calculate the redness index, greenness index and blueness index values for identifying digital leaf and flower images^14^. Thus, we can extract colour based features from shell images similarly for shell classification. In this study, we generated colour features from a colour histogram^15^ in the red, green and blue channels of a shell image (Fig. 3). For one shell sample, there are two colour images taken at different views. Therefore, we can generate two 256*3 (where 256 represents the number of grayscales ranging from 0 to 255) matrixes for the first shell image and second shell image respectively, then combine them together to construct a 256*6 matrix. The black background colour in a shell image was analysed by the flood fill algorithm^16^, which generated a corresponding black background mask for each shell image. Therefore, the corresponding black background colour in a shell image can be eliminated by calculating the number of black background mask pixels, which maintains the effectiveness of the extracted shell colour feature. The mean (*μ*) and standard deviation (*s*) of the intensity values for the red, green and blue channels were calculated from this generated 256*6 matrix. Thus, 12 elements were used for delegating the shell colour feature (each colour channel would generate two values: mean and standard deviation respectively). Therefore, we are able to classify shell species by using this extracted data effectively.

### Shape feature extraction

Numerous works can be found in literature discussing the extraction of shape features in plant leaf recognition. One of them is the widely used Centroid Contour Distance (CCD)^17^. This method is able to find out the distance between the centroid point and the boundary point, which is useful to extract the targeted object outside boundary.

**Fig. 4:**
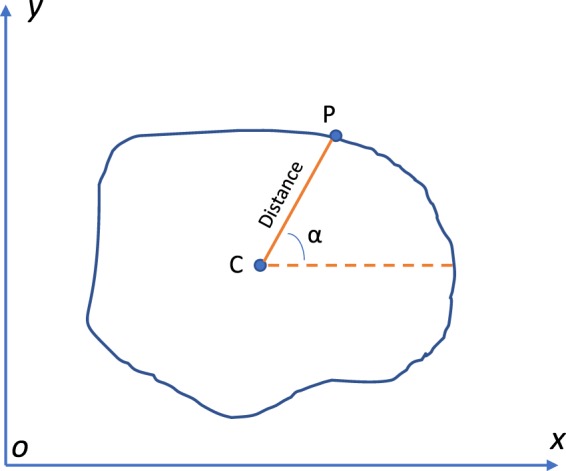
The principle of centroid contour distance.

CCD can trace a targeted object contour by circling around its centroid (Fig. 4). The midpoint C can be regarded as the object’s centroid, while P is one of the points on the boundary. The distance between the point P and the central point C is considered as the centroid-distance. When point P moves on the boundary of the object based on the angle α, the centroid-distance also changes. We can collect the various centroid-distances of one object based on the different angles, which are treated as the shape feature.

**Fig. 5:**
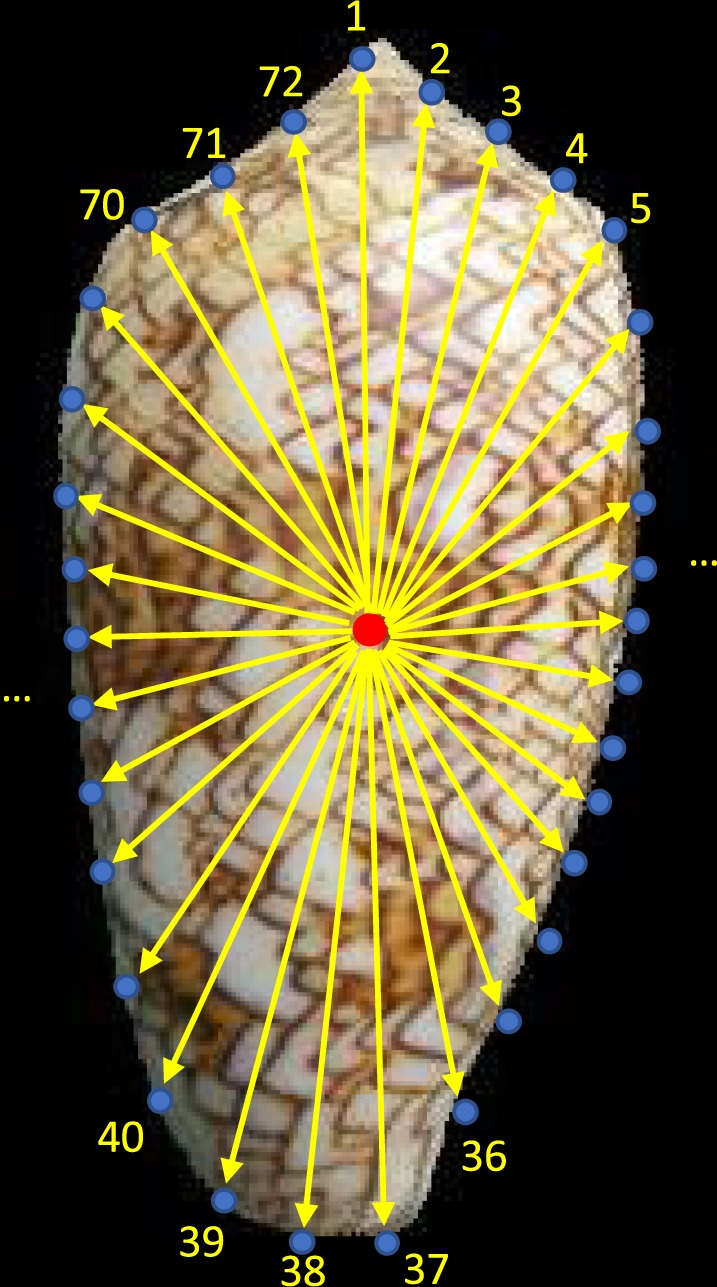
The number of boundary points based on an interval angle of α = 5°. The red point is this shell image’s central point, the blue points are obtained by using an interval angle of α = 5°, and the distances between the central point and boundary points are calculated and regarded as the shape feature of a shell.

In this article, we used the CCD method to extract the shape feature of shell images. The shell image was converted to grayscale, where we applied a flood fill algorithm^16^ to detect the black background part and generate to corresponding a background mask. The black background mask was inverted to generate a targeted shell mask of each shell image. Therefore, the boundary of a shell can be obtained from the shell mask and further processed by the CCD method. In Fig. 5, the central point (red point) can be calculated from the shell’s boundary (https://www.mathworks.com/help/matlab/ref/polyshape.centroid.html). And the point P (blue point from 1 to 72) on the shell’s boundary from 0° moves to 360° every step by 5° (using an interval angle of α = 5°, totally 360/5 = 72 steps), before calculating the distance between the central point and boundary point in every step, finally generating 72 distance points for one shell image.

### Texture feature extraction

Different shells have different decorative patterns on their surfaces, which can be considered as its texture feature in shell classification. The Gabor filter^18^ is a linear filter widely used in texture analysis and recognition, showing its potential performance^19^. In this section, a 2-D Gabor filter is applied to the grayscale shell images to generate its texture feature, which is given by Eqs (1) and (2):

1 $$f\left(x,\,y,\,\omega ,\,{\sigma }_{x},\,{\sigma }_{y}\right)=\frac{1}{2\pi {\sigma }_{x}{\sigma }_{y}}exp\left[\frac{-1}{2}({\left(\frac{x}{{\sigma }_{x}}\right)}^{2}+{\left(\frac{y}{{\sigma }_{y}}\right)}^{2}+j\omega (x{\rm{\cos }}\theta +y{\rm{\sin }}\theta ))\right]$$

2 $$r\left(x,y\right)=I(x,\,y)\times f\left(x,\,y,\,\omega ,\,{\sigma }_{x},\,{\sigma }_{y}\right)$$

where *σ* is the spatial width, *ω* is the frequency, and *θ* is the orientation. The different orientations (*θ*) and frequencies (*ω*) are key parameters in texture analysis and extraction. The *I(x, y)* in Eq. (2) indicates the grayscale image of a shell texture, *f*(*x*, *y*, *ω*, *σ*_*x*_, *σ*_*y*_) is the Gabor filter with different settings in the frequencies and orientations, and *r(x, y)* denotes the image filtered results by the Gabor filter.

There are different justifications for the choice of frequency (*ω*) and orientation (*θ*). Jain *et al*.^19^, used only four orientations (θ°, 45°, 90°, 135°) to reduce the computational cost, and selected frequencies based on psy-chophysic studies. The previous work by Recio *et al*.^20^, applied a set of frequencies and orientations which are determined empirically. In Cope *et al*.’s work^21^, the frequencies of the Gabor filter were chosen from 0 to 7. Based on the work of others and our many experiments, four different orientations (θ°, 45°, 90°, 135°) with five different frequencies (*ω* = 5, 10, 15, 20, 25) were chosen for the Gabor filter settings. Thus, we totally have 20 different Gabor filters to analyse the shell texture. As the computational cost and time for extracting the texture feature by applying Gabor filters is very high, we choose the first image (frontal view) of all shell samples for texture analysis. For each shell sample, 200 small patches (each patch is sized 20*20 pixel) from the surface of the shell were randomly selected, then transformed to greyscale images. Next, 20 different Gabor filters were applied to these 200 small patches in one shell sample, generating 4000 responses. Each response was then calculated to produce 3 features via the following equations:

3 $${\rm{Average}}\,{\rm{value}}:\,\sum _{(i,j)\in W}\frac{{r}_{ij}}{\left|W\right|}$$

4 $${\rm{Energy}}:\,\sum _{(i,j)\in W}\frac{{r}_{ij}^{2}}{\left|W\right|}$$

5 $${\rm{Entropy}}:\,-\sum _{\left(i,j\right)\in W}\frac{\left|{r}_{ij}\right|}{\left|W\right|}log\frac{\left|{r}_{ij}\right|}{\left|W\right|}$$

where *W* is the current patch, *r*_*ij*_ is the response for the current filter at pixel *(i, j)*, and $\left|W\right|$ is the number of pixels in each small patch.

After applying the aforementioned texture extractors, each shell sample forms a 4000*3 matrix. Here, the values of each row of this matrix were arranged from small to large, which is regarded as texture feature. As the size of the preliminary texture feature is too large to deal with, the principal component analysis (PCA) was applied to reduce the dimension of the shell texture matrix while preserving the features that contribute most to the variance in this dataset^22^. As over 95% of the variance in the dataset come from the first ten projected features, we utilize them as the texture feature. Hence, each shell sample texture is quantized by PCA to a 10 elements predetermined vector, which is considered as the final texture feature of a shell.

## Data Records

**Fig. 6:**
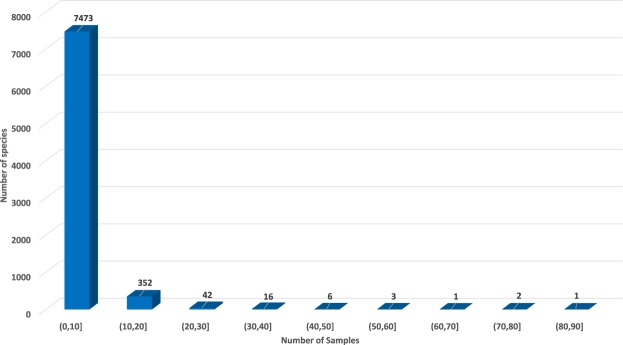
The distribution of sample numbers of all shell species. This dataset is a highly imbalanced shell data with 7894 species. Most of the shell species have less than 10 samples, while a few of them have over 30 samples.

A shell statistics figure has been plotted to show the shell sample distribution of the entire dataset (Fig. 6). The reorganized shell images dataset contains 29622 samples, where the complete 59244 images are available from the file all_shell_images_2nd.zip and can be downloaded. The sample number of each shell species is recorded as the file all_shell_species_inventory_revised.xlsx and is also available for download. All three features of the shell samples are extracted and recorded as files all_color_features, all_shape_features, and all_texture_features respectively, which are also available for download. 134 shell species’ images are analysed by three (colour, shape and texture) feature extraction methods, which is available as the file shell_species_134_data.zip. The colour feature extracted by the aforementioned colour feature extraction method (refer to Method: Colour feature extraction section), is also available as the file colour_feature_raw.xlsx, where post-processing by calculating the mean (*μ*) and standard deviation (*s*) can be found in the file colour_feature_processing.xlsx. Both are available for download. The shape feature extracted via the shape feature extraction method (see Method: Shape feature extraction section), is also available as the file shape_feature.xlsx ready for download. The texture feature extracted using the texture feature extraction method (refer to Method: Texture feature extraction section), is accessible as the file texture_feature_raw.xlsx along with its post-processing by PCA as the file texture_feature_processing.xlsx. Both are available for download. The complete shell dataset is openly available at the figshare repository^23^.

## Technical Validation

### Shell database

In order to prove the effectiveness and potentiality of the shell dataset, the extracted shell features were applied by two different classifiers: *k*-NN and random forest for shell recognition. It could be noted that this shell dataset is strongly unbalanced in terms of samples per shell species in Fig. 6. Thus, we choose all shell species (totally 134 species) with 10 samples (Online-only Table 1) in this study to validate the fairness and effectiveness of extracted features of this shell dataset. Afterwards, a total of 1340 samples were chosen for validation. The F1-score and accuracy of shell recognition is 78.23% and 77.39% respectively, when applying *k*-NN with the three features. Therefore, this proposed dataset can be considered as a robust and effective approach for shell species classification.

### K-nearest neighbours

**Fig. 7:**
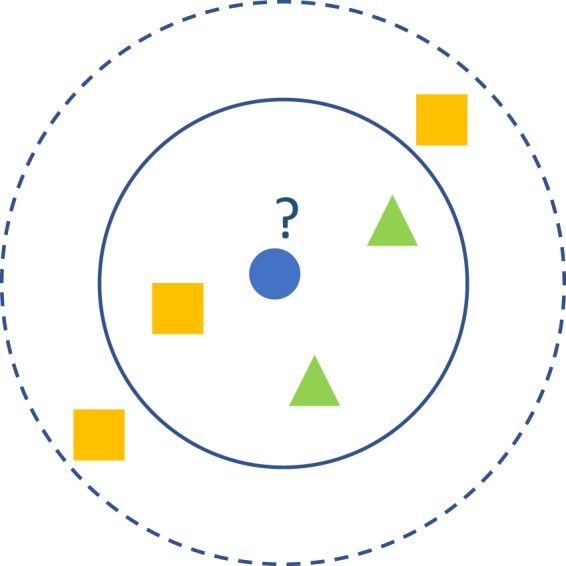
The illustration of *k*-NN classification. The test sample (blue circle) would be categorized to the first class of green triangles when *k* = 3 (solid line circle), as there are 2 green triangles and only 1 orange square inside the inner circle. However, it would be assigned to the second class of orange squares when *k* = 5 (dashed line circle), since there are 3 orange squares and only 2 green triangles inside the outer circle.

*k*-NN is a commonly used supervised learning method. Its working principle is very simple: it attempts to find the nearest *k* training samples in the training dataset based on a distance measurement, allowing it to predict the results using the information of these *k* neighboured samples in a testing dataset. The voting method usually can be applied in classification tasks, which generally chooses the most class markers in the *k* training samples as the prediction result^8^. Fig. 7 shows the schematic diagram of *k*-NN, where it is obvious to see that the *k* value is an important parameter. The classification results would be significantly different with different *k* values settings. In this validation, we investigated different *k* values to assess their performances for shell recognition and found the best classification results using the *k*-NN method.

### Random forest

The random forest is a classifier containing multiple decision trees in the training dataset, and its output class is determined by the mode of the classes of the individual trees^9^. It is based on a decision tree with bagging, which is further introduced with random attribute selection during the training process. Specifically, it randomly selects a subset containing *k* attributes for each node of the sub-decision trees, before choosing the best attribute from this subset for partitioning. Random forest is a simple, easy to implement and low computational method, showing good performances in many practical tasks. Here, we implemented random forest for shell recognition to evaluate the usability of this dataset.

### F1-score

To take the disparity of the samples in each class into account, we use an additional F1-score metric to perform evaluation^24^. Since F1-score is a compound of precision and recall, we utilize it as a comprehensive metric to provide performance evaluation.

The F1-score is described as follows:

6 $$F1-score=\frac{2{\sum }_{i=1}^{n}T{P}_{i}}{2{\sum }_{i=1}^{n}T{P}_{i}+{\sum }_{i=1}^{n}F{P}_{i}+{\sum }_{i=1}^{n}F{N}_{i}}$$

where *n* represents the number of classes, and *TP*_*i*_, *TP*_*i*_, *FP*_*i*_, *FN*_*i*_ are true positive, true negative, false positive and false negative of the *i* th class, respectively.

### Experiments

The two introduced classifiers were applied to evaluate this shell dataset. In particular, each extracted feature from a shell was first assessed individually. The parameters in the classifiers such as *k* were fine-tuned in order to obtain the best results. This process was repeated 30 times randomly, and the average of the 30 runs was used as final results. Next, three features from a shell were combined together to construct a vector with 166 dimensions, which was also evaluated by *k*-NN and random forest. The procedures of parameter selection, the proportion (70%) of the training dataset and the number of repeating times are the same to the individual extracted feature.

### Experimental results

**Fig. 8:**
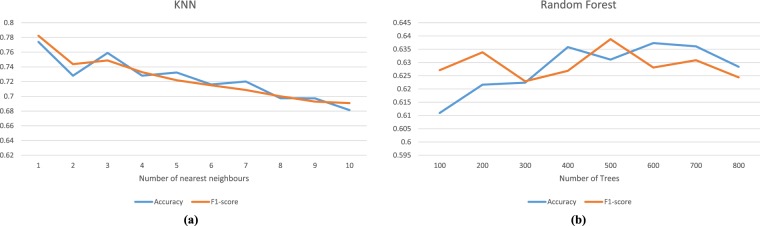
The performance of different *k* values and tree numbers in *k*-NN and random forest classification. The performance of different *k* values and tree numbers in *k*-NN and random forest classification. (**a**) presents the accuracy of shell recognition related to the *k* nearest neighbour number for *k*-NN using three features, while (**b**) shows the accuracy of shell recognition related to the tree number in random forest when using three features.

Tables 1–4 shows the F1-score and classification accuracy results of using shell colour feature, shape feature, texture feature and the combination of the three features with a confidence level (α = 95%) respectively. It should be noted that the shape feature is the most effective characteristic in shell classification, followed by the colour and texture features. When combining the three features, the final result is much better than any single feature, proving the validity of this shell dataset. Figure 8(a) shows the F1-score and accuracy related to the *k* value selection for *k*-NN (when three features are used simultaneously). The highest performance is reached when *k* is 1, which is regarded as the final classification result. In Fig. 8(b), F1-score and accuracy do not achieve their highest value with the same parameter using random forest. Here, we select the parameter with the highest accuracy, which is T = 600 as the final classification result. The F1-score and accuracy of shell recognition is 78.23% and 77.39% respectively, when applying *k*-NN with the three features (Table 4). Therefore, this proposed dataset can be considered as a robust and effective approach for shell species classification.

**Table 1:** Shell classification performance using colour feature.

Classifiers	F1-score (%)	Accuracy (%)
*k*-NN (*k* = 1)	50.35 ± 0.0149	50.87 ± 0.0132
Random forest	34.88 ± 0.0079	36.09 ± 0.0126

**Table 2:** Shell classification performance using shape feature.

Classifiers	F1-score (%)	Accuracy (%)
*k*-NN (*k* = 1)	67.66 ± 0.0130	66.71 ± 0.0134
Random forest	59.32 ± 0.0115	58.11 ± 0.0116

**Table 3:** Shell classification performance using texture feature.

Classifiers	F1-score (%)	Accuracy (%)
*k*-NN (*k* = 1)	15.17 ± 0.0113	16.39 ± 0.0090
Random forest	15.92 ± 0.0094	17.85 ± 0.0082

**Table 4:** Shell classification performance using 3 features.

Classifiers	F1-score (%)	Accuracy (%)
*k*-NN (*k* = 1)	78.23 ± 0.0119	77.39 ± 0.0107
Random forest	62.81 ± 0.0126	63.73 ± 0.0098

## Usage Notes

We provide the code of extracting the three features for shell images (can be found in the Code availability section). In addition, the features of 134 shell species with 10 samples were extracted and analysed by two classifiers *k*-NN and random forest in the technical validation section, which can be found in github: https://github.com/zqplus/shell-recognition/blob/master/ReadMe_how%20to%20generate%20shell%20features%20%26%20load%20data%20for%20classification/Shell_env. Researchers can directly use post-processing shell features data to find more effective machine learning methods for improving the performance of shell recognition, or attempt to investigate new algorithms to deal with shell species with small samples, even present new feature extraction methods for shell recognition based on the collected shell images.

## Data Availability

The code for extracting the three features from a shell can be found here: https://github.com/zqplus/shell-recognition/tree/master/ReadMe_how%20to%20generate%20shell%20features%20%26%20load%20data%20for%20classification.

## Online-only Table

**Online-only Table 1:** The number of shell species with a variety of number of samples that are evaluated in the technical validation section.

Shell name	Sample number	Shell name	Sample number	Shell name	Sample number
Aandara consociata	10	Acteon nakayamai	10	Aculamprotula fibrosa	10
Alia unifasciata	10	Amoria maculata	10	Amoria molleri	10
Ampeliata gaudens	10	Amphidromus dubius	10	Amphidromus elviae	10
Amphidromus rottiensis	10	Anachis paessieri	10	Anachis paessleri	10
Archachatina marginata	10	Argonauta argo	10	Batillaria multiformis	10
Bellamya species	10	Bistolida diauges	10	Blasicrura subteres	10
Bolinus brandaris	10	Bractechlamys vexillum	10	Bradybaena sequiniana	10
Bradybaena tourannensis	10	Brotia costula	10	Buccinanops moniliferum	10
Bullina virgo	10	Cantharus cecillei	10	Carpiscula procera	10
Caryocorbula contracta	10	Cellana tramoserica	10	Chicoreus cornucervi	10
Chione tumens	10	Cingulina species	10	Circe scripta	10
Clypidina notata	10	Cochlodina laminata	10	Conus capitaneus	10
Conus dusaveli	10	Conus milneedwardsi	10	Conus wakayamaensis	10
Corculum	10	Cosmetalepas concatenatus	10	Cristaria tenuis	10
Cryptonemella producta	10	Cuneopsis pisciculus	10	Cyclophorus clouthianus	10
Dentarene sarcina	10	Dioryx swinhoei	10	Diplommatina futilis	10
Domiporta filaris	10	Drupa morum	10	Duplicaria kieneri	10
Echinolittorina aspera	10	Elliptio congaraea	10	Ellipto lanceolata	10
Erosaria beckii	10	Erosaria guttata	10	Erronea onyx	10
Eucrassatella cumingii	10	Euphaedusa cetivora	10	Euphaedusa porphyrea	10
Faunus ater	10	Fusconaia subrotunda	10	Gudeodiscus phlyarius	10
Gyliotrachela muangon	10	Haliotis varia	10	Hemiphaedusa wenderi	10
Hemiplecta sibylla	10	Hemitrochus gallopavonis	10	Ischnochiton elongatus	10
Ischnochiton virgatus	10	Jenneria pustulata	10	Jullienia crooki	10
Lacunopsis coronata	10	Laeocathaica christinae	10	Lambis lambis	10
Leporicypraea geographica	10	Lirophora latilirata	10	Lithasia obovata	10
Littoridina parchappei	10	Lunella granulata	10	Lyncina lynx	10
Nanina citrina	10	Naria miliaris	10	Nassarius comptus	10
Obba listeri	10	Ocinebrellus inornatus	10	Oliva incrassata	10
Oliva jaspidea	10	Oliva lacanientai	10	Pachydrobia prasongi	10
Paphia textile	10	Paraprososthenia hanseni	10	Paraprososthenia levayi	10
Patelloida latistrigata	10	Peristernia nassatula	10	Petraeomastus xerampelinus	10
Phaedusa praecelsa	10	Phasianella solida	10	Phasianella variegata	10
Phenacovolva fusula	10	Philippia oxytropis	10	Phyllonotus pomum	10
Planorbis planorbis	10	Pleuroploca granosa	10	Pollicaria mouhoti	10
Potamocorbula amurensis	10	Pseudanachis duclosianus	10	Pseudotalopia sakuraii	10
Pupinidius melinostoma	10	Pupinidius porrectus	10	Pupopsis gansuicus	10
Rabdotus dealbatus	10	Rhynchotrochus woodlarkianus	10	Rissoina bruguieri	10
Scutus unguis	10	Septaria porcellana	10	Sinum javanicum	10
Smaragdia rangiana	10	Solen grandis	10	Stahylaea limacina	10
Strombus gibberulus	10	Subzebrinus ottonis	10	Tellina radiata	10
Tomigerus pilsbryi	10	Trachycardium pristipleura	10	Trochomorpha xiphias	10
Truncatellina species	10	Tucetona canoa	10	Vepricardium coronatum	10
Vexillum cadaverosum	10	Vexillum coronatum	10	Vexillum species	10
Zelippistes excentricus	10	Zeuxis dorsatus	10		
